# Anti-HIV Activity of Cucurbitacin-D against Cigarette Smoke Condensate-Induced HIV Replication in the U1 Macrophages

**DOI:** 10.3390/v13061004

**Published:** 2021-05-27

**Authors:** Sunitha Kodidela, Namita Sinha, Asit Kumar, Santosh Kumar

**Affiliations:** The Department of Pharmaceutical Sciences, College of Pharmacy, University of Tennessee Health Science Center, Memphis, TN 38163, USA; nsinha2@uthsc.edu (N.S.); akumar23@uthsc.edu (A.K.)

**Keywords:** Cucurbitacin-D, HIV, blood–brain barrier model, cytokines/chemokines, p24, macrophages, cigarette smoke condensate

## Abstract

Chemodietary agents are emerging as promising adjuvant therapies in treating various disease conditions. However, there are no adjuvant therapies available to minimize the neurotoxicity of currently existing antiretroviral drugs (ARVs). In this study, we investigated the anti-HIV effect of a chemodietary agent, Cucurbitacin-D (Cur-D), in HIV-infected macrophages using an in-vitro blood–brain barrier (BBB) model. Since tobacco smoking is prevalent in the HIV population, and it exacerbates HIV replication, we also tested the effect of Cur-D against cigarette smoke condensate (CSC)-induced HIV replication. Our results showed that Cur-D treatment reduces the viral load in a dose-dependent (0–1 μM) manner without causing significant toxicity at <1 μM concentration. Further, a daily dose of Cur-D (0.1 μM) not only reduced p24 in control conditions, but also reduced CSC (10 μg/mL)-induced p24 in U1 cells. Similarly, Cur-D (single dose of 0.4 μM) significantly reduced the CSC (single dose of 40 μg/mL)-induced HIV replication across the BBB model. In addition, treatment with Cur-D reduced the level of pro-inflammatory cytokine IL-1β. Therefore, Cur-D, as an adjuvant therapy, may be used not only to suppress HIV in the brain, but also to reduce the CNS toxicity of currently existing ARVs.

## 1. Introduction

The prevalence of HIV-associated neurocognitive disorders (HAND) is increasing despite the successful implementation of antiretroviral therapy (ART) [1,2]. There is an evidence of transmigration of CD14^+^CD16^+^ monocytes in the central nervous system (CNS), which perpetuates the neuropathogenesis of HIV [3]. However, there is a lack of association between neurocognitive impairment and virological and immunological indicators, which suggests the progression of neuronal damage in HIV-positive subjects regardless of the success of ART [4]. Furthermore, the detection of viral RNA is observed in the brains of subjects with complete suppression of plasma viral load by ART [5]. The increased viral load in the brain is mainly due to the inability of the current ART drugs to cross the blood–brain barrier (BBB) and suppress the virus efficiently [6,7]. The presence of even very low levels of viral replication in the CNS could result in neural injury or dysfunction due to prolonged exposure to inflammatory responses and neurotoxic viral proteins.

Cigarette smoking is prevalent among HIV-positive subjects [8] and it has been reported to increase HIV replication and its associated conditions by various mechanisms, including oxidative stress [9,10,11]. Furthermore, smokers with HIV are more likely to have a poorer response to ART and subsequently to develop more serious comorbidities and premature death than non-smokers with HIV [12,13]. Moreover, smoking was associated with a higher risk of progression from asymptomatic neurocognitive impairment to symptomatic HAND in people living with HIV [14]. Although the cessation of cigarette smoking is the ultimate cure for smoking-exacerbated HIV complications [12], it is difficult to achieve. Furthermore, the current use of ART drugs is ineffective against the virus in the brain, and it can induce neurotoxicity [15]. Recently, FDA has approved the first long-acting drug combo for HIV, monthly shots which are beneficial for people who are more likely to have a reduced compliance to the treatment, including people who smoke cigarette [16]. However, relatively high costs of the monthly shots may limit their use among the HIV population. Besides, this drug formulation does not solve the problem of ineffective treatment of HIV in the brain. Thus, there is a need to constantly develop new drugs or drug-like compounds that can, alone or in the presence of current ART drugs, effectively suppress HIV, as well as smoking-exacerbated HIV replication and its associated complications, including HAND. 

Naturally occurring dietary compounds have gained increasing attention for the prevention of various types of cancers and viral infections, including HIV [17,18,19,20]. Cucurbitacin-D (Cur-D) is a tetracyclic triterpene commonly found in the cucurbitaceae family, which has been used in conventional medicine for decades [17,21]. However, no study has demonstrated its anti-HIV activity. In this study, we show, for the first time, the potential anti-HIV activity of Cur-D across the in-vitro BBB model in the absence and presence of tobacco constituents.

## 2. Materials and Methods

### 2.1. Chemicals and Reagents

The cigarette smoke condensate (CSC) was purchased from Murty Pharmaceuticals (Lexington, KY, USA). The Cur-D was obtained from Sigma-Aldrich (St. Louis, MO, USA). The HIV Type 1 p24 Antigen ELISA kit to assess HIV viral load was purchased from ZeptoMetrix Corporation (Buffalo, NY, USA). Pierce Lactate Dehydrogenase (LDH) Cytotoxicity Assay Kit was purchased from ThermoFisher Scientific (Grand Island, NY, USA). Cell culture reagents including the Roswell Park Memorial Institute (RPMI) 1640 media were purchased from Corning Inc. (Tewksbury, MA, USA). Dulbecco’s Modified Eagle’s Medium (DMEM) was obtained from American Type Culture Collection. Fetal bovine serum (heat-inactivated) was bought from Atlanta Biologicals (Atlanta, GA, USA). L-glutamine and penicillin-streptomycin (P/S) were purchased from Fisher Scientific.

### 2.2. Cell Culture and Treatment

U1 cells, which are U937 cells chronically infected with the human immunodeficiency virus type 1 (HIV-1), were procured from the NIH AIDS Reagent Program (Germantown, MD, USA). The U1 cell lines have been widely used by our group and many other research groups to study the role of drug abuse, including tobacco smoking in HIV replication [10,11,22,23,24,25]. Furthermore, the results obtained using these cells have been successfully replicated in HIV-infected primary macrophages [10,11,24]. The U1 cells were cultured in RPMI 1640 media supplemented with 10% fetal bovine serum (FBS), 1% L-glutamine, and 2% penicillin/streptomycin. To differentiate the cells into macrophages, 0.3 million cells were seeded in 0.4 mL of media containing 100 nM phorbol 12-myristate 13-acetate) in each well of a 12-well plate. After 3 days of differentiation, the media was removed, cells were washed with phosphate buffer saline, and the fresh media was added to the differentiated cells. After adding the fresh media to the cells, the cells were incubated for 3–4 h before starting the treatment. The differentiated cells were treated with control (DMSO), CSC (10 μg/mL), Cur-D (0.1 μM), and CSC (10 μg/mL) + Cur-D (0.1 μM) every day for 3 days. In a separate experiment, we also used established ART drug regimen darunavir-ritonavir (DRV-RTV) (12 μg/mL and 4 μg/mL, respectively) as positive control and compared the data with Cur-D. We used DRV as a positive control because the literature suggests that DRV was detectable in cerebrospinal fluid samples of HIV subjects [26]. The p24 antigen levels were determined in the collected culture supernatant using the p24 ELISA kit. 

We procured mouse and human brain endothelial cells (CRL-2299) and astrocytes (CRL-2541) from ATCC (Manassas, VA, USA). These cells are validated by the ATCC and have been widely used to prepare the in vitro BBB model, including by our group [25]. These cells were cultured in DMEM media supplemented with 10% FBS and 1% PS solution and incubated at 37 °C with 5% CO_2_ before building in vitro BBB model. 

#### In Vitro BBB Model

We used Transwell^®^-COL collagen-coated 0.4 μm pore polytetrafluoroethylene membrane insert (Sigma-Aldrich) to prepare an in vitro BBB model as described previously [27].

Mouse model: First, we used mouse endothelial and astrocytic-cells to represent our future proposed work with the HIV mice model to study the pharmacokinetics, tissue distribution, and efficacy of Cur-D on viral suppression. Briefly, the mouse astrocytes (2 × 10^5^ cells/well) were seeded on the bottom of 12-well plates. After 24 h of adhesion, mouse endothelial cells (2 × 10^5^ cells/well) were seeded onto the upper side of the Transwell^®^- COL inserts, and the inserts were placed in a 12-well plate containing astrocytes. These cells constitute the BBB model and were grown for 5 days to achieve ~90% confluency. After achieving 90% confluency, the upper inserts containing endothelial cells were transferred to the wells containing U1-differentiated macrophages. Transendothelial electrical resistance (TEER) using EVOM2 Epithelial Voltohmmeter (World Precision Instruments, Sarasota, FL) was measured as described [27]. A mean TEER value of 100 to 120 Ohms × cm^2^ was observed in the confluent BBB model and published in our previous reports [27]). To determine the efficacy of Cur-D on CSC-induced viral replication, endothelial cells in the upper inserts were exposed to a single dose of control (DMSO), CSC (40 μg/mL), Cur-D (0.4 μM), and CSC (40 μg/mL) + Cur-D (0.4 μM) and observed for 3 days. In this case, we used a higher dose of CSC, because a lower CSC dose shows inability to cross the BBB and effectively suppress HIV across the BBB. HIV-1 viral loads were measured every day in the cell culture supernatant from the bottom chamber using a p24 ELISA kit.

**Figure 1:**
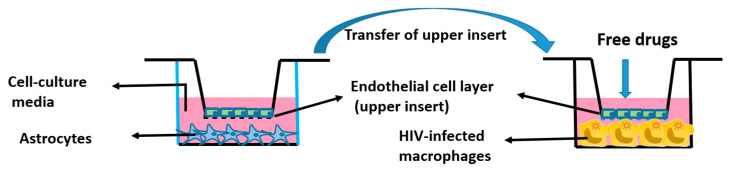
Graphical representation of the in vitro human BBB model.

Huma model: After establishing the effect of Cur-D against CSC-induced HIV replication in the mouse BBB model, we developed a human BBB model (Figure 1) to mimic the human system. In this model, we tested the anti-HIV effect of Cur-D and compared it with established ART regimen (DRV-RTV) as a positive control. For this, we used human astrocytes and endothelial cells and followed the same procedure mentioned above to develop a human BBB model. We treated the endothelial cells in the upper inserts with a single dose of control (DMSO), CSC (40 μg/mL), Cur-D (0.4 μM), CSC (40 μg/mL) + Cur-D (0.4 μM), and DRV-RTV (12 μg/mL and 4 μg/mL, respectively) and observed for 2 days. The human BBB model was relatively sensitive than the mouse BBB model, and thus we could only continue the treatment for up to 2 days.

### 2.3. HIV Type 1 p24 ELISA

The HIV-1 p24 antigen level in the supernatant collected from U1 cells was measured using an HIV-1 p24 Antigen ELISA kit (Zeptometrix Corporation, Buffalo, NY, USA). The kit consists of microwells that are coated with a monoclonal antibody specific for HIV-1 P24 antigen. The samples containing the HIV-1 p24 antigen were added to these wells. The captured viral antigen was sequentially incubated with biotin-labeled human antibody to HIV-1 for 1 h, streptavidin conjugated to horseradish peroxidase for 30 min at 37 °C, and tetramethylbenzidine substrate for 30 min in the dark. The optical density of every well was measured at 450 nm and compared against the standard curve to determine the p24 (pg/mL) levels in the samples. The viral load was expressed as a percentage of HIV-1 P24 levels observed in DMSO-treated control wells.

### 2.4. LDH Cytotoxicity Assay

Cytotoxicity was measured in the cell culture supernatant collected from differentiated U1 macrophage media using the Pierce Lactate Dehydrogenase (LDH) Cytotoxicity Assay Kit (ThermoFisher Scientific, Grand Island, NY, USA) following the manufacturer’s protocol. Briefly, 50 μL of the collected culture supernatant was mixed with 50 μL of the LDH reaction mixture in a 96-well plate and incubated at room temperature for 30 min. Then, the 50 μL of LDH stop solution were added to stop the reaction. The absorbance was measured at 490 nm and 680 nm using a microplate reader (Cytation™ 5 Cell Imaging Multi-Mode Reader, BioTek, VT, USA). Higher absorbance suggests higher toxicity. 

### 2.5. Western Blotting

To determine the expression of IL-1β, catalase, and SOD1, an equal amount of protein (10 μg) was used from control (DMSO), CSC, and Cur-D treated differentiated U1 macrophages. The proteins from different study groups were loaded onto polyacrylamide gel (4% stacking, 10% resolving gel), run for 90 min at 150 V, and then transferred to polyvinyl fluoride membrane using a current of 0.35 Amp for 90 min. After the proteins were transferred to the membrane, it was incubated with 5–10 mL of Li-Cor blocking buffer (LI-COR Biosciences, Lincoln, NE, USA) for 1 h to avoid the nonspecific binding of antibodies to its surface. The membrane was then incubated overnight at 4 °C with target primary antibodies (IL-1β Rabbit Pab, 1:500 dilution, proteintech, catalog#16806-1-AP; SOD1 mouse Mab, 1:200 dilution, Santa Cruz Biotechnology, catalog #sc-101523; Catalase mouse Mab, 1:100 dilution, Santa Cruz Biotechnology, catalog # sc-365738; β-Actin Mouse mAb.1:2000 dilution, Cell Signaling, Catalog #3700) at 4 °C overnight. The next day, the blots were washed with PBS containing 0.2% Tween-20 (PBST) three times and then incubated with the corresponding secondary antibodies (Goat anti-Mouse Mab, Goat anti-Rabbit Mab, 1:10,000 dilution, LI-COR Biosciences) for 1 h at room temperature in the dark. The membrane was washed again with PBST and the blots were scanned using Image Studio Lite version 4.0 in a Li-Cor Scanner (LI-COR Biosciences). The densitometric data was obtained from the Image Studio Lite software. Actin was used as an internal loading control to normalize the expression of IL-1β, catalase, and SOD1 proteins.

### 2.6. Cytokine Analysis

The protein levels of various cytokines and chemokines such as pro-inflammatory: TNF-α, IL-1β, IL-8, IL-6; anti-inflammatory: IL-1ra, IL-10; and chemokines: MCP-1, and RANTES were measured from the culture media (25 μL) of differentiated U1 macrophages using Human Custom Procartaplex 8-plex (Invitrogen, ThermoFisher Scientific, Grand Island, NY, USA). Following the manufacturer’s protocol, samples, standards, and magnetic beads were added to the 96-well ELISA plate and mixed well on a plate shaker for 1 h at room temperature, followed by overnight incubation at 4 °C. The next day, the beads were washed, followed by the addition of the detection antibody, streptavidin-PE, and reading buffer, with subsequent washing off of reagents at each step. The concentration (pg/mL) of the cytokines and chemokines were measured using a Magpix system, and the data were analyzed using the xPONENT^®^ software.

### 2.7. Statistical Analysis

The GraphPad Prism 5 (GraphPad Software; La Jolla, CA, USA) was used to perform all statistical analyses and to plot graphs. The data are presented as mean ± SEM. One-way ANOVA with Tukey’s post-hoc test was applied to compare between multiple groups; *p* < 0.05 is considered significant. 

## 3. Results

### 3.1. Cur-D Does Not Exhibit Cytotoxicity in U1 Macrophages

**Figure 2:**
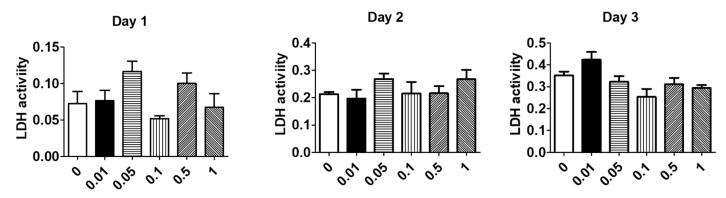
Effect of different doses of cucurbitacin-D (Cur-D) on cytotoxicity of U1 macrophages: U1-monocytes were plated in 12-well plates (0.3 million cells/well) and differentiated to macrophages. Differentiated U1 macrophages were treated with different doses of Cur-D ranging from 0.01–1 μM daily for 3 days. The Lactate Dehydrogenase (LDH) release in the supernatant was measured every day by the LDH assay.

Since there is a lack of data on the safe dose of Cur-D in U1 macrophages, we performed an LDH assay to analyze the cytotoxicity of Cur-D on U1 cells. For this, we treated U1 cells with different concentrations of Cur-D (0, 0.01, 0.05, 0.1, 0.5, and 1 μM) every day for 3 days. We observed that treatment of U1 cells with 0.01–1 μM of Cur-D for 1, 2, and 3 days did not show a statistically significant increase in LDH activity (Figure 2), suggesting no detectable cytotoxicity with the selected doses. There appears to be an inconsistent pattern of toxicity on day 1, perhaps due to the initial stress caused by the treatment, which is a common observation with treatment with any xenobiotic agent. 

### 3.2. Treatment with Cur-D Reduces p24 Levels in U1 Cells

**Figure 3:**
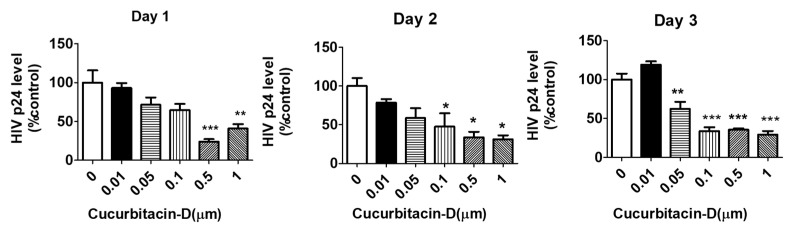
Dose-dependent effect of Cur-D on HIV p24 levels: U1-monocytes were plated in 12-well plates (0.3 million cells/well) and differentiated to macrophages. Differentiated U1 macrophages were treated with different doses of Cur-D ranging from 0.01–1 μM for 3 days. The p24 levels were measured every day by the p24 Elisa kit. Viral load was expressed as a percentage of viral load observed in DMSO-treated control wells. The data shown represent the mean ± SEM of three independent experiments. One-way ANOVA with Tukey’s post-hoc test was applied to compare between multiple groups. *, **, and *** represent *p* < 0.05, *p* ≤ 0.01, and *p* ≤ 0.001, respectively, when compared to 0 μM of Cur-D.

To determine the anti-HIV activity of Cur-D, we treated U1 macrophages with 0.01–1 μM of Cur-D every day for 3 days. We observed a dose-dependent reduction in the viral load with Cur-D treatment in 1 and 2 days (Figure 3). Treatment with 0.1, 0.5, and 1 μM for 2 and 3 days showed a significant reduction in the viral load. There was no significant difference in the reduction of viral load between 0.1 vs. 0.5 μM and 0.1 vs. 1 μM of Cur-D in 3 days. Therefore, we selected 0.1 μM of cur-D for the subsequent experiments.

### 3.3. Relative Anti-HIV Effect of Cur-D Compared to DRV/RTV Positive Control

**Figure 4:**
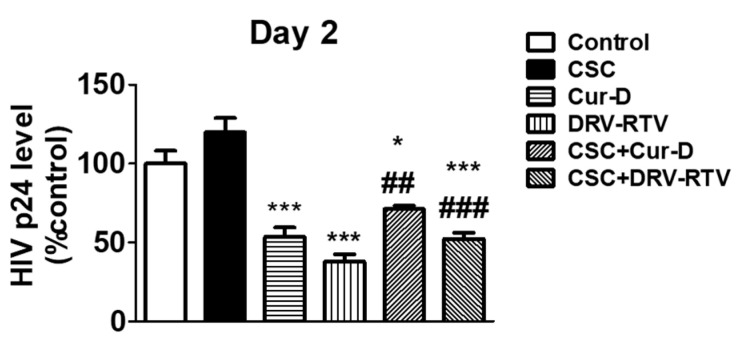
Comparison of the anti-HIV effect of Cur-D with darunavir/ritonavir (DRV/RTV): To compare the anti-HIV effect of Cur-D with DRV/RTV on cigarette smoke condensate (CSC)-induced viral replication, differentiated U1 macrophages were treated with Control (DMSO), CSC (10 μg/mL), Cur-D (0.1 μM), and DRV-RTV (3 μg/mL/1 μg/mL) and observed for 2 days. HIV viral loads from media of U1 cells were measured on day 2, using a p24 ELISA kit. One-way ANOVA with Tukey’s post-hoc test was applied to compare between multiple groups. * and *** represent *p* < 0.05, and *p* ≤ 0.001, respectively, when compared to control. ## and ### represent , *p* ≤ 0.01 and *p* ≤ 0.001, respectively when compared to CSC.

After establishing the anti-HIV effect of Cur-D at different doses and time points, we tested whether the anti-HIV activity of Cur-D is comparable to that of established ART drugs, with DRV-RTV as a positive control. To test this, we treated U1 macrophages with 0.1 μM of Cur-D and 3 μg/mL of DRV (C_max_ of oral 400 mg QD), 1 μg/mL of RTV (C_max_ value of oral 100 mg QD) with and without CSC (10 μg/mL) for 2 days. After 2 days of treatment, p24 levels were measured in the collected media. As expected, both Cur-D and DRV-RTV showed a significant reduction in p24 levels compared to both control and CSC (Figure 4). Although not significant, the reduction in viral load with Cur-D was slightly lower than with DRV-RTV. Thus, compared to the established ART regimen, Cur-D also was effective against HIV replication alone as well as in the presence of CSC (Figure 4).

### 3.4. Treatment with Cur-D Decreases CSC-Induced HIV Replication

**Figure 5:**
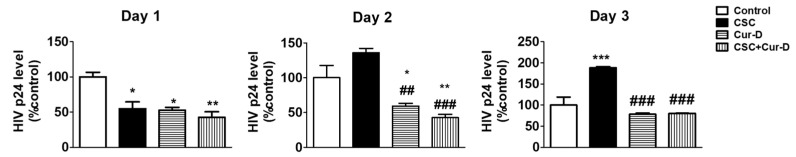
Effect of Cur-D on CSC-induced HIV replication: Differentiated U1 macrophages were concomitantly treated with CSC (10 μg/mL) and Cur-D (0.1 μM) every day for 3 days. HIV p24 levels were measured at the end of the treatment using an ELISA kit. One-way ANOVA with Tukey’s post-hoc test was applied to compare between multiple groups. *, **, and *** represent *p* < 0.05, *p* ≤ 0.01, and *p* ≤ 0.001, respectively when compared to control. ##, and ### represent *p* ≤ 0.01, and *p* ≤ 0.001, respectively when compared to CSC.

After establishing the anti-HIV effect of Cur-D alone as well as in the presence of CSC, we investigated the time-dependent effect of Cur-D on CSC-induced HIV replication. To determine this, we treated U1 macrophages concomitantly with CSC (10 μg/mL) and Cur-D (0.1 μM) every day for 3 days. As expected, except on day 1, CSC increased p24 in 2 and 3 days of treatment in U1 differentiated macrophages. Furthermore, we observed that treatment with 0.1 μM Cur-D reduced CSC-induced p24 on both day 2 and day 3 (Figure 5). 

### 3.5. Cur-D Reduces a Major Pro-Inflammatory Cytokine, IL1β, in HIV Infected Macrophages

**Figure 6:**
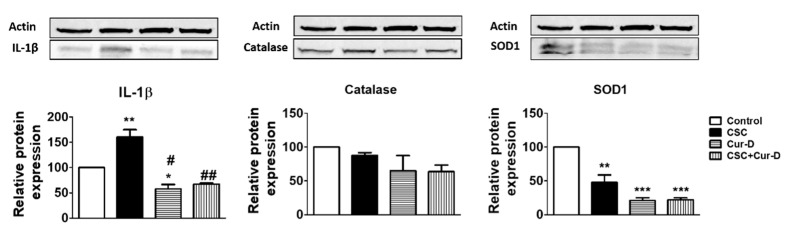
Effect of Cur-D on IL-1β and antioxidant enzymes (catalase and SOD1): Differentiated U1 macrophages were concomitantly treated with CSC (10 μg/mL) and Cur-D (0.1 μM) continuously for 3 days and cells were harvested at the end of the treatment. The expression of IL-1β, antioxidant enzymes (catalase and SOD1) proteins were measured in differentiated U1 cells (*n* = 3) by Western blot. One-way ANOVA with Tukey’s post-hoc test was applied to compare between multiple groups. *, **, and *** represent *p* < 0.05 and *p* < 0.01, *p* < 0.001, respectively when compared to control. # and ## represent *p* < 0.05 and *p* < 0.01, respectively, when compared to CSC.

Cucurbitacins have been shown to exert antioxidant and anti-inflammatory properties [28]. Therefore, we investigated whether the anti-HIV effect of Cur-D is mediated through antioxidant or anti-inflammatory pathways. We observed that a key pro-inflammatory cytokine, IL1β, which is known to play a major role in HIV disease progression [29,30], is significantly increased with CSC exposure (Figure 6). Upon Cur-D treatment, the levels of IL1β were significantly decreased. Furthermore, we observed that the Cur-D treatment significantly reduced the levels of the antioxidant enzyme, SOD1, while showing a pattern of reduction in the levels of catalase (Figure 6). 

### 3.6. Cytotoxicity of CSC and Cur-D in U1 Cells after Crossing the In Vitro Mouse BBB Model

**Figure 7:**
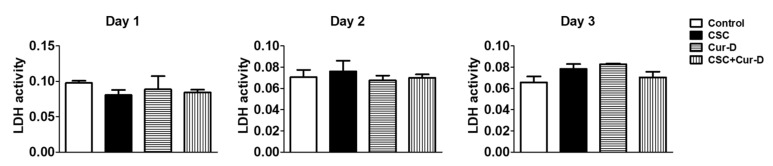
Toxicity of CSC and cur-D in U1 cells after crossing the in vitro blood–brain barrier (BBB) model: To determine the toxicity of CSC and Cur-D across the BBB, we used U1 cells to create a modified in vitro BBB model in a Transwell^®^ plate, as described in the methodology. The upper inserts containing endothelial cells were exposed to a single dose of Control (DMSO), CSC (40 μg/mL), and Cur-D (0.4 μM), and toxicity from differentiated U1 macrophages present in the lower wells were measured every day for 3 days, using an LDH assay kit from the culture media of the bottom chamber. One-way ANOVA with Tukey’s post-hoc test was applied to compare between multiple groups.

To determine the toxicity of CSC and Cur-D across the BBB, we used mouse endothelial and astrocytic cells to form a BBB layer and used U1 cells to create a modified in vitro BBB model in a Transwell^®^ plate, as described in the Methods section. We used mouse endothelial and astrocytic cells to represent our proposed future work using the HIV mice model to study the pharmacokinetics and pharmacodynamics of Cur-D. The upper insert of the transwell system had confluent endothelial cells and the lower chamber had differentiated U1 macrophages. The upper inserts were exposed to one dose of control (DMSO), CSC (40 μg/mL), and Cur-D (0.4 μM), and toxicity was measured every day for 3 days, using the LDH assay kit from the culture media of the bottom chamber containing U1 cells. We observed that the treatment with CSC and Cur-D did not induce any cellular toxicity (Figure 7).

### 3.7. Treatment with Cur-D Decreases CSC-Induced HIV Replication across the Mouse BBB Model

**Figure 8:**
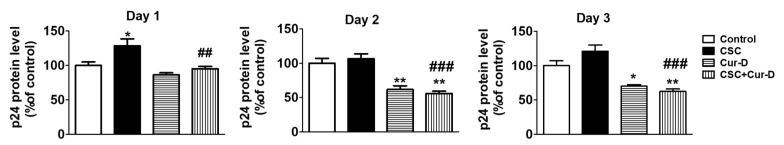
Effect of CSC and Cur-D on the viral load after crossing the in vitro BBB model: To determine the efficacy of Cur-D on CSC-induced viral replication, we used differentiated U1 cells to create a modified in vitro BBB model in a Transwell^®^ plate, as described in the methodology. The upper inserts containing endothelial cells were exposed to a single dose of Control (DMSO), CSC (40 μg/mL), and Cur-D (0.4 μM) and observed for 3 days. HIV-1 viral loads from differentiated U1 cells were measured every day, using a p24 ELISA kit from the culture media of the bottom chamber. One-way ANOVA with Tukey’s post-hoc test was applied to compare between multiple groups. * and ** represent *p* < 0.05 and *p* ≤ 0.01, respectively, when compared to control. ## and ### represent *p* ≤ 0.01 and *p* ≤ 0.001, respectively when compared to CSC.

To determine whether Cur-D can reduce the CSC-induced HIV replication in the CNS, we concomitantly treated differentiated U1 cells with one dose of Cur-D (0.4 μM) and CSC (40 μg/mL) across the BBB model. We measured P24 levels every day in the medial collected from U1 cells. Although the effect was variable, we observed that CSC, even in the in vitro BBB model, showed increased viral replication at all the time points (day 1–3) (Figure 8). Furthermore, we observed that Cur-D could significantly reduce the CSC-induced HIV replication in both day 2 and day 3 treatment.

### 3.8. Changes in Pro-Inflammatory and Anti-Inflammatory Cytokines with Exposures of CSC and Cur-D to U1 Cells across the Mouse BBB Model

**Figure 9:**
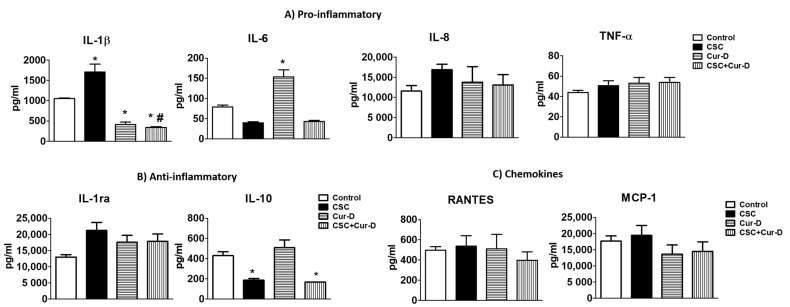
Measurement of cytokines in U1-cell media treated with CSC and Cur-D across the BBB model: The upper inserts containing endothelial cells were exposed to a single dose of Control (DMSO), CSC (40 μg/mL), and Cur-D (0.4 μM) and observed for 3 days. The protein levels of various cytokines and chemokines were measured from the culture media of the bottom chamber containing differentiated U1 macrophages using Human Custom Procartaplex 8-plex. One-way ANOVA with Tukey’s post-hoc test was applied to compare between multiple groups. * represents *p* < 0.05 when compared with control. # represents *p* < 0.05 when compared with CSC.

Since the IL-1β level was significantly elevated with direct exposure to CSC, and the IL1-β level was reduced by Cur-D treatment (Figure 6), we investigated whether other cytokines and chemokines were also altered. Many cytokines and chemokines have been reported to be altered in HIV and HIV-positive smokers [9,31]. Therefore, we examined eight cytokines/chemokines across the BBB model (Figure 9). As observed with direct exposure, CSC significantly elevated IL-1β whereas treatment with Cur-D significantly reduced its level even in the presence of CSC across the BBB model. On the other hand, treatment with Cur-D elevated IL-6 level, which is known to play a dual role during infections [32,33,34,35]. However, Cur-D did not cause an elevation in IL-6 level in the presence of CSC. Furthermore, exposure of Cur-D to U1 cells did not significantly increase IL-10 levels compared to control whereas CSC exposure significantly reduced it (Figure 9). Finally, we did not observe a significant difference in the levels of chemokines with either CSC or Cur-D exposure to U1 cells (Figure 9).

### 3.9. Treatment with Cur-D Decreases CSC-Induced HIV Replication across the Human BBB Model

**Figure 10:**
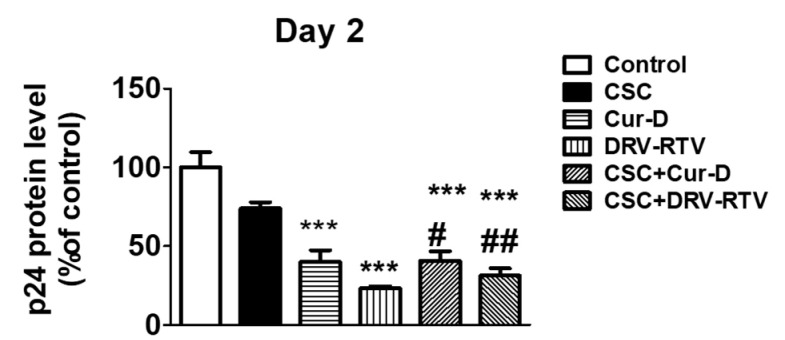
Effect of CSC and Cur-D on the viral load after crossing the in vitro human BBB model: To determine the efficacy of Cur-D on CSC-induced viral replication, we used differentiated U1 cells to create a modified in vitro human BBB model in a Transwell^®^ plate, as described in the methodology. The upper inserts containing endothelial cells were exposed to a single dose of Control (DMSO), CSC (40 μg/mL), Cur-D (0.4 μM), and DRV-RTV (12 μg/mL and 4 μg/mL, respectively) and observed for 2 days. HIV viral loads from U1 cells were measured every day, using a p24 ELISA kit from the culture media of the bottom chamber. One-way ANOVA with Tukey’s post-hoc test was applied to compare between multiple groups. *** represents *p* ≤ 0.001when compared to control. #, and ## represent *p* < 0.05, and *p* ≤ 0.01, respectively, when compared to CSC.

After observing the effect of Cur-D on CSC-induced HIV replication in the mouse BBB model, we further established a human BBB model using human endothelial and astrocyte cell lines. We observed that the human BBB model is relatively more sensitive, and thus the treatment lasted for only two days. Briefly, the upper inserts containing human endothelial cells were exposed to one dose of control (DMSO), CSC (40 μg/mL), Cur-D (0.4 μM), and DRV-RTV (12 μg/mL and 4 μg/mL, respectively) for 2 days. We measured p24 levels in the media collected from U1 cells on Day 2. Although CSC did not show an effect, we observed that both Cur-D and DRV-RTV significantly reduced the viral load compared to control (Figure 10). Importantly, both Cur-D and DRV-RTV reduced CSC-induced HIV replication on Day 2, with DRV-RTV showing a relatively higher effect than Cur-D. Thus, the findings showed a similar effect of Cur-D and DRV-RTV on HIV replication in both the human and mouse BBB models.

## 4. Discussion

Various chemodietary agents and nutraceuticals have been shown to be effective against several types of cancers and infectious diseases [17,18,19,20,36,37,38]. Among these, curcumin has been tried as adjuvant therapy in clinical studies to treat various conditions largely for cancers [39,40,41]. Curcumin has also been shown to possess anti-HIV activity [19,20,42]. However, its limited bioavailability impedes its use for therapeutic applications [43,44]. Although cucurbitacins, especially Cur-D, have also been studied for cancer treatments as adjuvant therapy [45,46,47], their use in the treatment of infectious diseases, especially HIV, is not studied. To the best of our knowledge, this is the first study to show the anti-HIV activity of Cur-D. One of the major limitations of the use of cucurbitacins is its relatively high toxicity profile [17,48,49]. However, the concentrations that we tested (<1-μM range) in the present study were found to be safe (Figure 2) and effective in suppressing the HIV replication directly (Figure 3) as well as across the BBB model (Figure 8 and Figure 10) in differentiated U1 macrophages. Furthermore, the anti-HIV effect of Cur-D was comparable to that of established ART regimen, DRV-RTV, in both the direct as well as BBB model experiments (Figure 4 and Figure 10). Thus, our study provides evidence that Cur-D at <1-μM range could be useful in suppressing HIV not only in the peripheral macrophages, but also in the CNS reservoirs such as brain perivascular macrophages and microglia.

Cigarette smoking is prevalent among HIV-positive subjects [8,50]. It can cause oxidative damage [9,10], which in turn can induce BBB damage leading to increased BBB permeability [51,52,53]. The increased BBB permeability augments infiltration of HIV-infected monocytes and influx of other peripheral toxins into the brain [51]. Consequently, it activates neuro-inflammatory pathways by increasing glial activation, leading to HAND [54]. Therefore, it is important to study the effect of Cur-D on CSC-induced HIV replication across the BBB model. We observed that treatment with Cur-D reduces p24 levels across the BBB model, suggesting that it can cross the BBB to a sufficient extent and suppress the HIV replication.

Chronic inflammation and immune suppression are the major hallmarks of HIV infection. Dysregulation of cytokine production has been shown to contribute significantly to HIV replication and disease progression [55,56,57]. Several reports suggest that HIV replication/disease progression is associated with elevated levels of IL-1β [29,30,58]. In the present study, the increased levels of IL-1β with CSC exposure suggest that CSC exacerbates HIV replication. The Cur-D treatment reduces IL-1β levels alone as well as in the presence of CSC exposure, suggesting that its anti-HIV activity is mediated through its anti-inflammatory pathway. Our findings are supported by those of Yang et al. who reported that Cur-D significantly suppressed the production of IL-1β in keratinocytes and thus may be useful as an anti-inflammatory agent for psoriasis [59]. Although Yoshida et al. suggest that Cur-D increases LPS induced Il-1β level, Cur-D alone did not elevate the IL-1β level in macrophages [60].

Interestingly, the IL-6 level was significantly decreased with the CSC exposure. Our results are supported by those of Zhao et al. who reported that CSC exposure significantly reduces the IL-6 secretion in mouse macrophage cell lines [61]. We also observed a similar trend in clinical samples in which the IL-6 level was relatively low in HIV subjects who smoke compared to HIV-positive subjects alone [31]. However, the exact mechanism by which CSC reduces the level of IL-6, a pro-inflammatory cytokine, is not clear. In the current study, treatment with Cur-D showed an increased level of IL-6. A study by Weimer et al. suggests that increased IL-6 secretion together with decreased IL-10 secretion appear to be involved in inducing CD4 helper dysfunction in HIV-positive subjects [62]. In their study, the authors have also observed that a patient who presented with increased IL-6 secretion, but no diminished IL-10 secretion, had a normal T-cell clone helper function. Moreover, the patient did not progress to developing AIDS during a 6-month observation period, despite an extremely low CD4 cell count of 45/μL. This suggests an important role of unaffected IL-10 secretion in a CD4 helper function. In our study, although the treatment with Cur-D increased IL-6 level, it did not significantly affect the IL-10 level, suggesting that increased IL-6 level with Cur-D might not contribute to CD4 cell dysfunction.

IL-10 is an important immunoregulatory cytokine with multiple biological effects. In the present study, the IL-10 level was significantly reduced with CSC exposure. These results are in line with our previous findings observed in plasma samples of HIV-positive smokers [31]. Said et al. reported that increased IL-10 production by monocytes is one of the mechanisms by which microbial products inhibit T-cell function in HIV-infected subjects [62]. Furthermore, IL-10 production is positively correlated with increased peripheral CD4+T cell depletion and increased numbers of microbes such as *M. tuberculosis* in HIV-positive subjects [63]. Overall, these findings suggest a positive correlation of IL-10 production with CD4 T cell dysfunction in HIV infection. In the present study, compared to control, the IL-10 level did not change with Cur-D treatment, suggesting that Cur-D may not cause T-cell dysfunction. To confirm this, we are in the process of developing an HIV-infected T-cell model.

The literature and our studies have shown the role of oxidative stress, generated by CSC, on HIV replication [9,10]. As expected, CSC reduced the levels of AOEs, especially SOD1, suggesting an increase in oxidative stress. However, Cur-D alone as well as in the presence of CSC also reduced the level of SOD1. The findings suggest that Cur-D does not suppress HIV, either directly or in the presence of CSC, via the oxidative stress pathway. On the other hand, a decreased level of SOD1 by Cur-D could be explained by its toxic nature, as Cur-D shows toxicity to many cells, especially to cancer cells [45,64,65]. In fact, due to its toxic role to kill cancer cells, Cur-D is studied to be used as adjuvant therapy in cancer treatment [45].

The major limitation of currently used ART drugs is their inability to cross the BBB and eliminate the virus from the brain [66,67]. Some of these ART drugs are also reported to cause neurotoxicity [15]. Thus, there is a need to use chemodietary agents as adjuvant therapy that help suppress the virus in the brain at relatively low and non-toxic ART drug concentration. To achieve the desired effects of ART drugs at low and nontoxic doses, Cur-D can be used as adjuvant therapy in HIV treatment. Although the use of adjuvant therapy is commonly implemented in cancer treatment to reduce the toxicity of chemotherapy, no drug has been tried for HIV treatment.

One of the limitations of our study is that we did not show the effect of Cur-D on T-cells, which are the central target of HIV therapies and HIV disease progression. Our goal in this project was to find a natural compound that can be used as an adjuvant therapy to suppress viral replication in the brain HIV reservoirs. It is well-documented that the HIV infection of the CNS results from the transmigration of infected CD4+ cells and monocytes across the BBB [68]. However, these HIV-infected cells entering the brain do not constitute an HIV reservoir since they have short half-lives [69,70]. Nevertheless, these cells, especially HIV-infected monocytes, are the source of the infection of three long-lived cell types: astrocytes, perivascular macrophages, and microglial cells. Since HIV infection in astrocytes appears to be non-productive, they cannot serve as HIV reservoirs. Therefore, the evidence suggests that both perivascular macrophages and microglial cells are susceptible to HIV infection and support productive infection. The productive infection in these cells in the brain has been associated with HAND in humans. Therefore, we selected macrophages for the study, as they are the cells of origin for perivascular macrophages and microglia in the CNS. Furthermore, the in vitro BBB models may not mimic the physiological BBB, because they lack three-dimensional structure and endothelial exposure to physiological shear stress, and restrict the maintenance of BBB properties in fully differentiated cells [71]. Therefore, the next goal is to test whether Cur-D can achieve therapeutic concentrations to suppress HIV in the periphery, particularly in T-cells, and in CNS cells. We will also test whether increased Cur-D concentration in the CNS suppresses HIV, as well as enhances the efficacy of ART drugs using both in vitro three-dimensional BBB and in vivo HIV animal models.

## 5. Conclusions

In conclusion, Cur-D reduces HIV replication directly as well as across the BBB models. It is also effective against CSC-induced HIV replication. Therefore, the present study provides the potential for Cur-D to be developed as adjuvant therapy in HIV treatment. It may be used not only to suppress HIV in the brain, but also to reduce the CNS toxicity of currently existing ART drugs. However, before we realize its role as an anti-HIV agent in adjuvant therapy, the detailed pharmacokinetics, tissue distribution, especially in the brain, and pharmacodynamic effects in an appropriate humanized HIV animal model are needed.
